# Novel microwire-based biosensor probe for simultaneous real-time measurement of glutamate and GABA dynamics in vitro and in vivo

**DOI:** 10.1038/s41598-020-69636-1

**Published:** 2020-07-29

**Authors:** P. Timothy Doughty, Imran Hossain, Chenggong Gong, Kayla A. Ponder, Sandipan Pati, Prabhu U. Arumugam, Teresa A. Murray

**Affiliations:** 1 Center for Biomedical Engineering and Rehabilitation Sciences, Louisiana Tech University, Ruston, LA USA; 2 Institute for Micromanufacturing, Louisiana Tech University, Ruston, LA USA; 3 UAB Epilepsy Center/Department of Neurology, The University of Alabama at Birmingham, Birmingham, AL USA

**Keywords:** Neural circuits, Neurological disorders

## Abstract

Glutamate (GLU) and γ-aminobutyric acid (GABA) are the major excitatory (E) and inhibitory (I) neurotransmitters in the brain, respectively. Dysregulation of the E/I ratio is associated with numerous neurological disorders. Enzyme-based microelectrode array biosensors present the potential for improved biocompatibility, localized sample volumes, and much faster sampling rates over existing measurement methods. However, enzymes degrade over time. To overcome the time limitation of permanently implanted microbiosensors, we created a microwire-based biosensor that can be periodically inserted into a permanently implanted cannula. Biosensor coatings were based on our previously developed GLU and reagent-free GABA shank-type biosensor. In addition, the microwire biosensors were in the same geometric plane for the improved acquisition of signals in planar tissue including rodent brain slices, cultured cells, and brain regions with laminar structure. We measured real-time dynamics of GLU and GABA in rat hippocampal slices and observed a significant, nonlinear shift in the E/I ratio from excitatory to inhibitory dominance as electrical stimulation frequency increased from 10 to 140 Hz, suggesting that GABA release is a component of a homeostatic mechanism in the hippocampus to prevent excitotoxic damage. Additionally, we recorded from a freely moving rat over fourteen weeks, inserting fresh biosensors each time, thus demonstrating that the microwire biosensor overcomes the time limitation of permanently implanted biosensors and that the biosensors detect relevant changes in GLU and GABA levels that are consistent with various behaviors.

## Introduction

Near real-time measurement of neurotransmitter levels in the brain is expected to reveal important clues to the underlying mechanisms of neurological disorders, such as epilepsy, addiction, motor control^1–3^, and secondary damage after stroke^4^ and traumatic brain injury^5,6^. Two important and ubiquitous neurotransmitters in the brain are l-glutamine (GLU) and γ-aminobutyric acid (GABA), which are the major excitatory and inhibitory neurotransmitters, respectively. A balance of excitatory (E) and inhibitory (I) electrical signaling in local networks (E/I ratio) is observed in awake states and in sleep, with the exception of slow-wave sleep in humans and monkeys^7^. Dysregulation in E/I is closely linked to dysregulation of GLU and/or GABA dynamics^8^. Prolonged, excessive GLU release often leads to excitotoxic neuronal cell death directly or through a cascade of secondary cellular injury^9,10^. Furthermore, insufficient release of GABA is associated with seizures^6,11^ and autism^12^. Most studies so far have only looked at GLU and GABA in isolation^13,14^. However, a more meaningful interpretation of the mechanisms of disease and secondary injury can be obtained by knowing the regional E/I balance^15^. Furthermore, the assessment of this E/I balance in different clinical, neurological and neuro-developmental disorders will help refine therapeutic electrical stimulation strategies in the future.

Excitatory and inhibitory synaptic inputs and intrinsic membrane properties determine the neuronal response (i.e., the output) to stimuli. The neuronal output, in the form of action potentials in different patterns and frequencies, propagates along the axons to produce local and distant neural effects^16^. Although diverse hypotheses exist to explain the therapeutic mechanisms of deep brain stimulation (DBS), most authors agree that DBS influences neuronal output and that frequency of stimulation is one of the crucial determinants of clinical outcome^17^. For example, clinical experience in epilepsy suggests that high frequency (> 140 Hz) stimulation reduces seizure frequency by decreasing neural excitability and desynchrony, while 50 Hz stimulation provokes seizures^18–20^. In Parkinson’s disease, stimulation below 50 Hz worsens symptoms, while temporally patterned stimulation like the theta-burst can improve memory^21–23^. Considerable work has attempted to delineate the cellular and network effects to bridge our understanding between the stimulation parameters and behavioral responses. However, to-date, mapping the dynamic changes in stimulation-evoked excitatory and inhibitory neurotransmitters simultaneously remains a technical challenge. Understanding of stimulation-induced changes in neurotransmitters is critical, as neurotransmitters modify synaptic strength and membrane excitability, and they shape neuronal connectivity that affects the downstream transmission of circuit-level information. For example, stimulation-induced changes in neurotransmitters have been associated with changes in the clinical outcome for subthalamic DBS in Parkinson’s disease^24^.

After the release of GABA and GLU, transporters on astrocytes and neurons in the tripartite synapse internalize the neurotransmitters which then convert the neurotransmitters to glutamine, and finally to GLU or GABA^25^. However, binding of these neurotransmitters to transporters is stochastic^26,^ and sufficient quantities diffuse away from the synapse to enable detection in the extracellular fluid^27^. Normally, local networks with inhibitory feedback tightly control release of these neurotransmitters^28,29^; however, this system appears to break down in some diseases and injury states^12,18,30^. Yet, the use of measurement tools with rates too slow to observe sub-second changes in extracellular GLU and GABA dynamics have likely contributed to research discrepancies^9,10,31^. One of the most common methods for the acquisition of longitudinal measurements is microdialysis, but analyte measurement is limited to large cell populations. In addition, its low sampling rate, on the order of minutes, prevents its use in measuring faster, real-time transients^2,3,31–35^, introducing a low-pass filtering effect into recorded data^36^. Precision, micromachined dialysis probes have increased the sampling rate to several seconds, but this resolution is still slow relative to the expected dynamics of GLU and GABA in the extracellular space. Furthermore, microdialysis appears to underreport neurotransmitter concentrations, possibly because artificial cerebral fluid is perfused into the dialysis cannula which dilutes the neurotransmitters^35,37^. Alternatively, enzyme-based biosensors based on microelectrode arrays (MEAs) may be able to improve spatial and temporal resolution, as well as biocompatibility^10,32^. For these types of biosensors, an analyte-specific enzyme is coated onto an electrode to selectively convert non-electrically-active molecules into substituent molecules, which can be oxidized or reduced further at the electrode to produce a measurable electric current that is proportional to the amount of analyte^32,34^.

While GLU microbiosensors can effectively measure real-time changes in extracellular GLU, real-time detection of GABA without the addition of exogenous substrates has only recently been developed^34^. In our previous work, we developed a shank-style microbiosensor array with enzymatic coatings to detect GABA and GLU^34,38^. Glutamate oxidase (GOx) immobilized on the surface of one of the probe sites facilitated the breakdown of non-electrically-active GLU into $\alpha$-ketoglutarate (α-keto), NH_3_ and hydrogen peroxide (H_2_O_2_, Eq. 1)^10,32,34,39^.

1 $${\text{GLU}}_{{({\text{E}})}} + {\text{H}}_{{\text{2}}} {\text{O}} + {\text{O}}_{{\text{2}}} \to \alpha {\text{-ketoglutarate}} + {\text{NH}}_{{\text{3}}} + {\text{H}}_{{\text{2}}} {\text{O}}_{{{\text{2}}({\text{E}})}}$$

2 $${\text{GABA}} + \alpha {\text{-ketoglutarate}} \to {\text{SSA}} + {\text{GLU}}_{{({\text{GABA}})}}$$

3 $${\text{GLU}}_{{({\text{GABA}})}} + {\text{H}}_{{\text{2}}} {\text{O}} + {\text{O}}_{{\text{2}}} \to {\alpha } {\text{-ketoglutarate}} + {\text{NH}}_{{\text{3}}} + {\text{H}}_{{\text{2}}} {\text{O}}_{{{\text{2}}({\text{GABA}})}}$$

Abbreviations are, as follow: GLU_E_ = environmental GLU, H_2_O_2(E)_ = H_2_O_2_ from GLU_E_, SSA = succinic semialdehyde, GLU_(GABA)_ = GLU from GABA oxidation, H_2_O_2(GABA)_ = H_2_O_2_ from GABA two-step reaction.

A + 0.7 V bias on the underlying platinum (Pt) microelectrode oxidized H_2_O_2_ molecules, releasing two electrons per H_2_O_2_ molecule that induced proportional and measurable current. This current was converted into a GLU concentration based on pre-calibration values^10^. The + 0.7 V potential helps to decrease noise by reducing interference from nuisance molecules that oxidize at higher potentials compared to hydrogen peroxide^3,31^. GABA detection is typically facilitated by the application of the GABASE enzyme to an electrode, but an exogenous application of α-keto was previously necessary to facilitate the breakdown of GABA to produce H_2_O_2_ (Eq. 2). While not a problem for in vitro studies, exogenous application of α-keto is unsuitable for in vivo studies. However, the product of the GOx reaction with GLU is α-keto (Eq. 3). To create our GABA microbiosensor, we applied both GABASE and GOx to one electrode to record the combined current. We then subtracted the current from an adjacent GOx-coated electrode to derive GABA current^34^.

The GOx enzyme produced a sufficient amount of α-keto at the GABA-GLU biosensor to support the conversion of GABA to H_2_O_2_ by GABASE. A third probe site (sentinel) was coated identically to the other sites except that no enzymes were used; this provided a means to measure and subtract signals from electrically active interferent molecules, such as ascorbic acid (AA). An additional size-exclusion layer of m-phenylenediamine (mPD), that rejects larger sized molecules, including most AA molecules^2,10,34,40^, was applied over the coated probe sites to mitigate the effect of interferents. Disadvantages of using extra coatings are the inhibition of the desired analyte’s diffusion and eventual breakdown of the coatings with continued use^40^.

A limitation of enzyme-based biosensors is that the enzyme coating is gradually degraded, reducing its effectiveness and eventually invalidating its readings. Dash et al*.* reported linearly decreasing amplitude over the first four days following implantation^31^, while Rutherford et al. reported consistently repeatable recording for 7 days after surgery, with one biosensor still usable at 23 days^3^. Our own experience with an in vivo model of status epilepticus revealed inconsistent results from permanently implanted enzyme-based, ceramic MEA probes and highlighted the need for a way to replenish or replace the enzyme layer over a long-term experiment (see Scoggin et al., Supplemental Data^38^).

To overcome the short probe lifespan caused by degradation of enzyme coatings of permanently implanted microbiosensors, we created a replaceable microwire biosensor. The GABA biosensor was based on our GLU and reagent-free GABA biosensor coated onto a shank-style probe, as previously reported^34^. The microwire biosensor was designed for insertion into a thin, permanently implanted cannula. The biosensor can be removed after recording and replaced with a filler wire to keep the cannula lumen clear until the next recording session. Then, a fresh biosensor is placed into the channel for the next recording session weeks or months after cannula implantation. This cannula protects the brain from the trauma of multiple insertions and ensures that recordings are made from the same population of neurons over time. We also used a “same-plane” geometry in which the ends of all three wires, which were the microsensor sites, terminate at the same depth in tissue. This geometry is well suited for use in rodent brain slice and cell culture models and for in vivo recording in brain regions with a laminar structure, such as the hippocampus and the neocortex. As a first test of the new biosensor, we used the same-plane geometry to measure real-time dynamics of GLU and GABA in rat hippocampal brain slices. We hypothesized that low-frequency electrical stimulation would result in higher E/I ratios than high-frequency stimulation^41,42^. Our results supported this hypothesis and revealed a linear increase in GABA that resulted in a nonlinear decrease in the E/I ratio as stimulation frequency increased. Toward our long-term goal of longitudinal, in vivo recording, we implanted a cannula into the hippocampus of a rat. We recorded from this awake and freely moving rat at 2, 10, and 16 weeks after cannula implantation using different microwire biosensors for each session. Unlike the large, prolonged release of neurotransmitters observed in electrically stimulated brain slices, in vivo peaks in GABA and GLU were lower, and changes were at time scales from less than 1 s to more than 8 min. After acquiring baseline recordings of an awake, behaving rat (Week 2), we induced status epilepticus which initiated epileptogenesis. As expected, this resulted in spontaneous seizures a few weeks later. We recorded again at Week 10 and Week 16, inserting freshly calibrated microwire biosensors into the implanted cannula for each session. GABA and GLU dynamics were consistent with reported changes in these neurotransmitters during behaviors, including grooming, walking, sleep–wake cycles^43–45^, and stages of seizure^7,46,47^. Our proof-of-concept procedure shows that the replaceable microwire biosensor overcomes the problem of sensor degradation common to permanently implanted biosensors. This device will better facilitate longitudinal studies of changes in GABA and GLU, along with behavior, in rodent models of neurological disorders and treatment.

## Results

### Microwire biosensor array characterization

**Figure 1:**
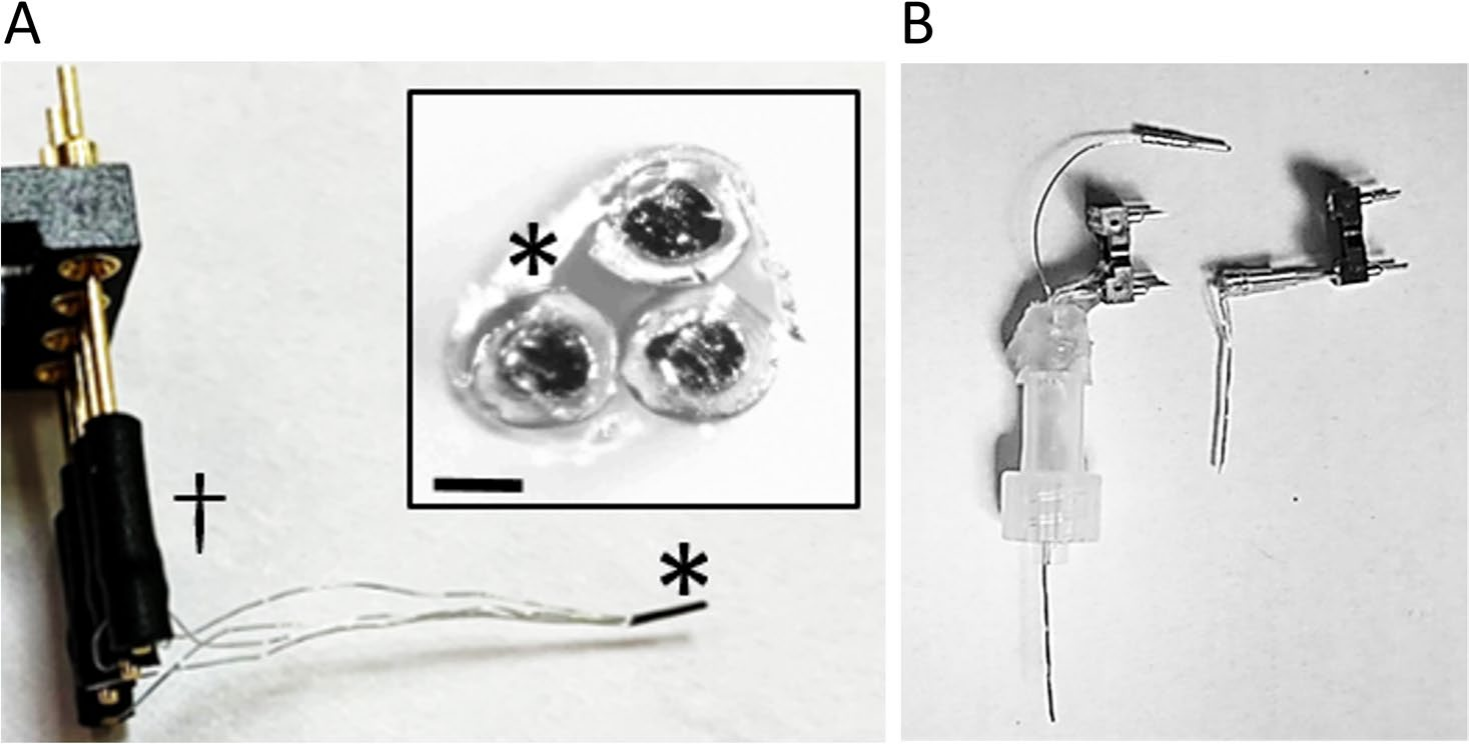
Assembled microwire biosensor. (**A**) The biosensor consists of three surface-modified platinum microwires that are packaged using heat shrink tubing (*). This simple and scalable packaging ensures close packing at the sensor side of the microwires and allows them to terminate in a single plane perpendicular to the sensor axis. Microwires were connected to pins of a Mill-Max connector using heat shrink tubing (dagger). This device was used for brain slice recordings. Inset. View of the distal, recording end of the biosensor. Scale bar denotes 100 µm. The ends of the coated microwires appear as dark circular areas surrounded by light-colored insulation; heat shrink tubing surrounds all three microwires (*). (**B**) Photograph of microwire biosensors for in vivo recording (left) and ex vivo recordings (right). For in vivo recordings, the microwire bundle at the distal end of the device is inserted into a permanently implanted cannula (not shown), and the translucent plastic Luer lock fitting is attached to the implanted cannula (Luer lock connector at bottom of fitting). A ground-connector wire runs through the fitting to connect a previously-implanted ground wire to the recording system. One end of the wire is looped inside the lumen of the Luer lock fitting in the screw connector (bottom of fitting) while the other end exits the top of the fitting, near the Mill-Max pins (single wire terminating with a pin connector). The loop of wire makes a connection with a matched loop of wire in the cannula connector. For in vitro and ex vivo recording, the device does not have a Luer lock fitting. For experiments, it is held in a micromanipulator by its Mill-Max connector. A separate ground wire (not shown) is placed away from the biosensor array.

**Figure 2:**
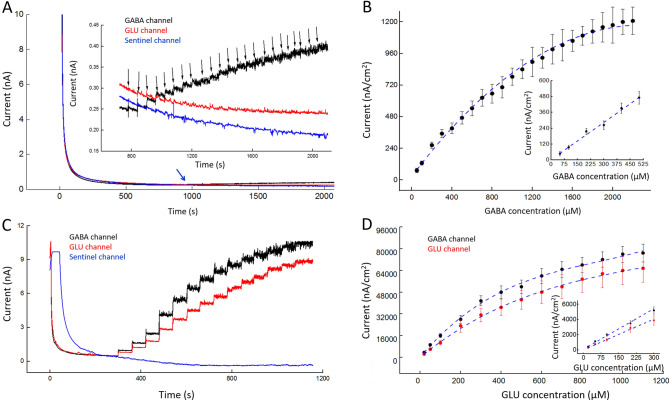
Characterization of GABA and GLU microwire biosensors. Measurements were acquired in 1X PBS containing 1 mM α-keto with a 200-rpm stirring rate at 37 °C. Amperometry parameters were + 0.7 V vs Ag/AgCl. (**A**) Representative plot of the response to GABA at the GABA (black), GLU (red), and sentinel (blue) microwire biosensors. **Inset A** Detail of (**A**) from 600 to 2,100 s. Black arrows correspond to the stepwise addition of GABA (50, 100, 200, 300, 400, 500, 600, 700, 800, 900, 1,000, 1,100, 1,200, 1,300, 1,400, 1,500, 1,600, 1,700, 1,800, 1900, 2000, 2,100, and 2,200 μM GABA respectively). As expected, corresponding stepwise increases in current occurred with increasing GABA concentration. The first two downward black arrows indicate addition of 50 μM GABA; this was followed by 20 subsequent additions of 100 μM GABA. GLU and sentinel channels were unaffected. (**B**) Current density at GABA biosensor increases with increasing concentration of GABA (mean ± SEM, n = 3). **Inset B** The linear response range is 0–500 μM GABA (slope of linear fit is 0.96 ± 0.15 nA/µM cm^2^, R^2^ = 0.982). Sensitivity in linear range is 1.2 ± 0.4 nA μM^−1^ cm^−2^. (**C**) Representative plot of the response to GLU at the GABA (black), GLU (red), and sentinel (blue) microwire biosensor. Both microwire biosensors show stepwise increases in current as GLU concentration increases (black downward arrows denote addition of GLU, 20, 50, 100, 200, 300, 400, 500, 600, 700, 800, 900, 1,000, 1,100, and 1,200 μM GLU respectively). (**D**) Current density at GABA and GLU biosensors increases with increasing concentrations of GLU (mean ± SEM, n = 3). **Inset D** The range of the linear response is 0–300 μM GLU. Sensitivity of GLU in the linear range is 144 ± 20 nA μM^−1^ cm^−2^ for the GABA microwire and 128 ± 25 nA μM^−1^ cm^−2^ for GLU microwire (mean ± SEM, n = 3 each, difference is not significant, *p* = 0.43).

A FAST-16MkIII potentiostat with an Ag/AgCl reference electrode and a + 0.7 V applied potential to each microwire biosensor were used to detect hydrogen peroxide generated by the enzyme-mediated breakdown of L-glutamate and GABA. Each microwire biosensor (Fig. 1) was calibrated using a range of concentrations of its analyte(s) (Fig. 2A, C). In addition, current density versus concentration was plotted, and then the linear range and sensitivity were determined for each microwire biosensor (described in Fig. 2B, D, n = 3). GABA biosensors had a linear range of 0–500 μM. GLU was detected on both the GABA and the GLU biosensors; their linear range for both was 0–300 μM. A slightly higher sensitivity for GLU was observed at the GABA biosensor than at the GLU biosensor (144 ± 20 nA/µM cm^2^ and 128 ± 25 nA/µM cm^2^, respectively; mean ± SEM, n = 3 each). This difference was not significant (*p* = 0.43). The selectivity against GABA is 29 ± 7. The selectivity against GLU is 123 ± 27 and 106 ± 21 for GABA and GLU channels, respectively. Selectivity was calculated as the sensitivity ratio of GLU or GABA to AA. AA had no effect on current, as seen in Supplemental Data (Fig. S2). Our sensitivity values compare well with the literature, which is predominantly based on planar arrays^48^. The linear and working ranges of the biosensors are well suited to measure their dynamics in the electrically stimulated slices studied in this work.

Selectivity was further improved through subtraction of the interferent signal from the “sentinel” microwire. The sentinel was prepared the same way as the GABA and GLU microwires, including mPD coating, except that the matrix coating on the sentinel contained only bovine serum albumin (BSA) and glutaraldehyde (i.e., it did not include an enzyme). BSA is an inactive protein of a size that is similar to GABASE and GOx; this maintains approximately the same rate of diffusion ^3,10,31,32,40^. Similar diffusion rates of interferent molecules at all three biosensors enables subtraction of sentinel current from GABA and GLU current to minimize the effect of interferents.

### Conversion of peak biosensor current responses to peak GLU and GABA concentrations

**Figure 3:**
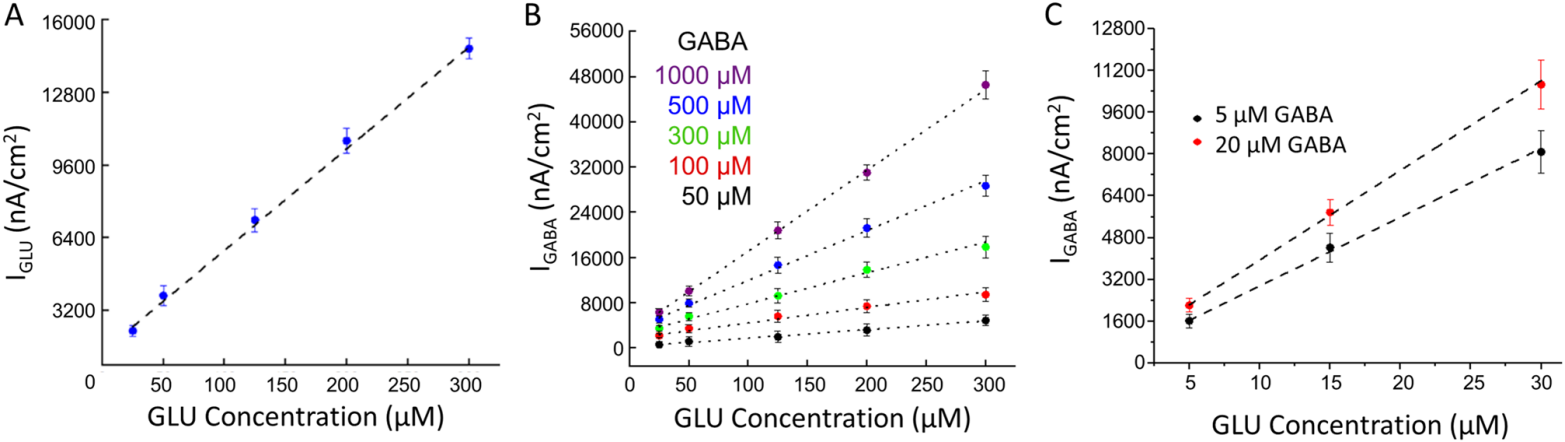
Representative calibration curves for microwire biosensors. Measurements were acquired in 1X PBS with a 200-rpm stirring rate at 37 °C. Amperometry parameters were + 0.7 V vs Ag/AgCl. (**A**) GLU calibration curve. This plot is typical of those used to determine GLU concentration from current measurements (slope = 44 ± 9 nA/µM cm^2^, R^2^ = 0.933, mean ± SEM, n = 3). (**B**) This set of calibration curves is typical of those used to determine GABA concentration (in μM) using the current, I_GABA_, at the GABA biosensor in a range of GABA concentrations (50–1,000 μM) in the presence of a range of GLU concentrations. Slopes (nA/µM cm^2^) and fits of curves for each GABA concentration are, as follows: 50 μM, 14 ± 4, R^2^ = 0.994; 100 μM, 28 ± 6; R^2^ = 0.975; 300 μM, 55 ± 14, R^2^ = 0.985; 500 μM, 89 ± 23, R^2^ = 0.994; 1,000 μM, 146 ± 32; R^2^ = 0.999 (mean ± SEM, n = 3 for each concentration). (**C**) Curves used to determine GABA concentration in a lower μM range (5–20 μM) in the presence of a range of GLU concentrations. Slopes and fits of curves for each GABA concentration are, as follows: 5 μM, 16 ± 2 nA/µM cm^2^, R^2^ = 0.999; 20 μM, 25 ± 3 nA/µM cm^2^; R^2^ = 0.996 (n = 3).

Calibration curves were created for each GLU and GABA biosensor channel (Fig. 3). The conversion of peak current to concentration began with the subtraction of the peak sentinel current (I_Sentinel_) from the raw peak current at the GLU channel (I_RAW_._GLU_) to derive the peak GLU current (I_GLU_) as shown in Eq. (4). The GLU current (I_GLU_ in pA) was then divided by the sensitivity of the GLU biosensor (SS_GLU_ in pA/μM) to produce the peak GLU concentration ([GLU] in μM, Eq. 5).

4 $${{\text{I}}_{{{{\rm GLU}}}} } = {\text{I}}_{{{{\rm RAW}}.{{\rm GLU}}}} - {\text{I}}_{{{{\rm Sentinel}}}}$$

5 $$\left[ {{\text{GLU}}} \right] = {\text{I}}_{{{\text{GLU}}}} /{\text{SS}}_{{{\text{GLU}}}}$$

6 $${\text{I}}_{{{\text{GLU}}.{\text{in}}.{\text{GABA}}}} = \left[ {{\text{GLU}}} \right] \times {\text{SS}}_{{{\text{GLU}}.{\text{in}}.{\text{GABA}}}}$$

7 $${\text{I}}_{{{\text{GABA}}}} = ({\text{I}}_{{{\text{GABA}} + {\text{GLU}}}} - {\text{I}}_{{{\text{GLU}}.{\text{in}}.{\text{GABA}}}} ) - {\text{I}}_{{{\text{Sentinel}}}}$$

The GABA channel detects both GABA and GLU. The current contributed by GLU in the GABA channel (I_GLU.in.GABA_, Eq. 6) is the peak concentration of the GLU channel ([GLU] in μM) multiplied times the sensitivity of GLU in the GABA channel when GABA concentration is 0 μM (SS_GLU.in.GABA_ in pA/μM). To determine the peak GABA current (I_GABA_), the current contributed by GLU (I_GLU.in.GABA_) was subtracted from the raw GABA channel current (I_GABA+GLU_) and the peak sentinel current was also subtracted (Eq. 7). The plot of I_GABA_ versus GLU concentration (Fig. 3B) was used to determine the concentration of GABA. For example, a peak I_GABA_ of 12,500 nA/cm^2^ with a peak GLU concentration of 130 μM would have a horizontal and a vertical line, respectively, that intersect just under the line for 500 μM GABA. The concentration of GABA is 481 μM.

### Extracellular GLU and GABA release in response to varied stimulation frequency

**Figure 4:**
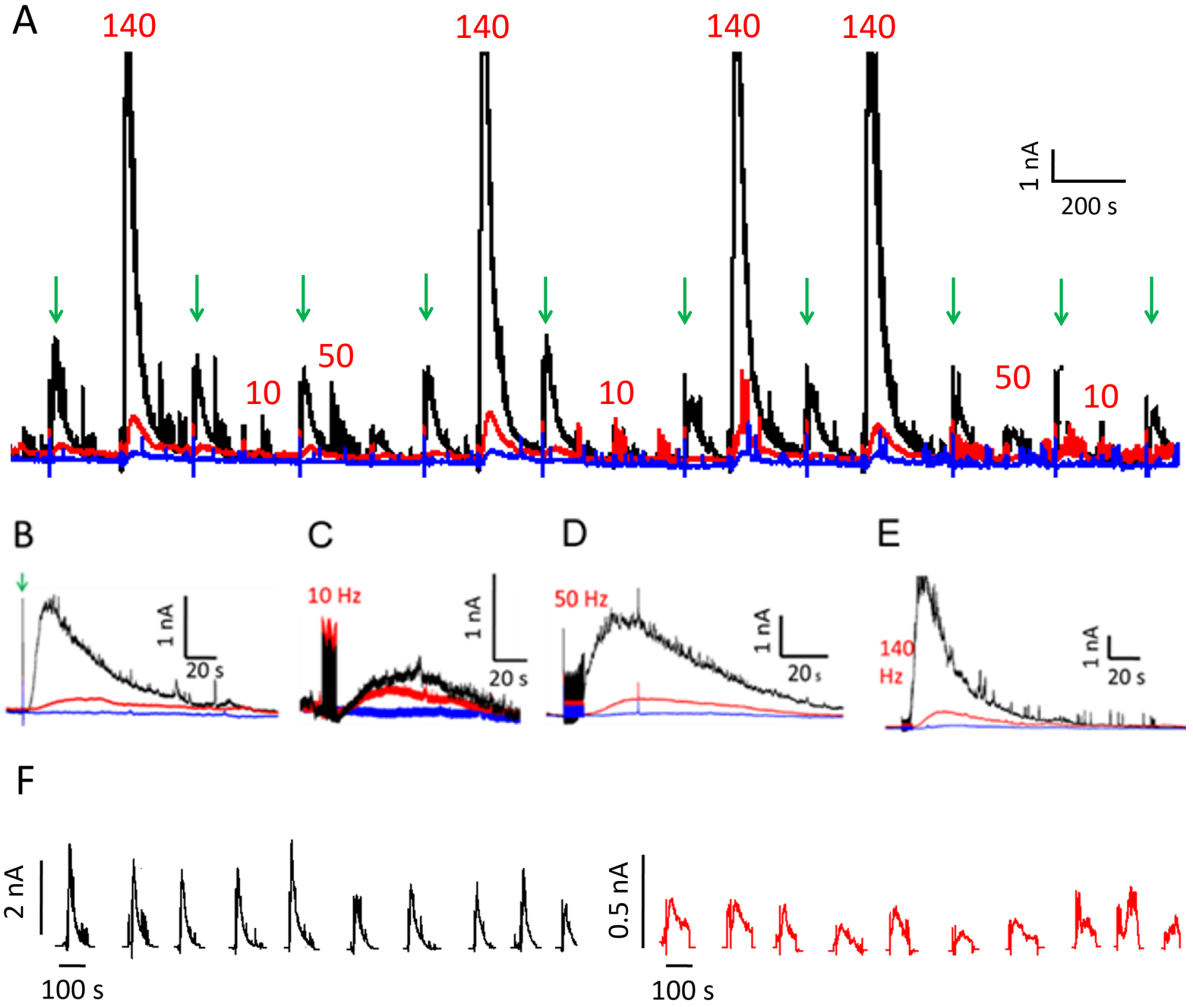
Microwire biosensor current in response to three different stimulation frequencies in brain slices. (**A**) Representative recording of current in response to 100-μA stimulation (GABA (black), GLU (red), and sentinel (blue)) with 10-, 50-, or 140-Hz pulse trains and to a single 100-ms control pulse indicated by green downward arrows. (**B**–**E**) Details of selected current responses to (**B**) control pulse, (**C**) 10-, (**D**) 50-, and (**E**) 140-Hz stimulation from Panel A. Densely spaced vertical lines preceding peaks are stimulation artifacts. (**F**) Consecutive responses to control pulses for recording in A (GABA, black; GLU, red).

We used 3 stimulation frequencies, 10, 50, and 140 Hz, to evoke GLU and GABA release. Each of these stimuli issued 5-s trains of 100-μA square pulses with 1 ms width^49–52^. We randomized the order of the three types of stimulations to avoid systematic error. Prior to initiating the next stimulation, we allowed current responses to return to baseline to avoid effects from potentiation. Between each stimulation, we used a single 100-ms control pulse (100-μA square pulse). Control pulses produced the same GLU response throughout each recording session, indicating that brain slices were in good health and that stimulation-induced potentiation had not occurred^53^. Both GABA and GLU current increased with increasing stimulation frequency. Representative traces of current responses to randomized stimulation are shown in Fig. 4.

### Extracellular concentration of GLU and GABA increases with higher stimulation frequency

**Figure 5:**
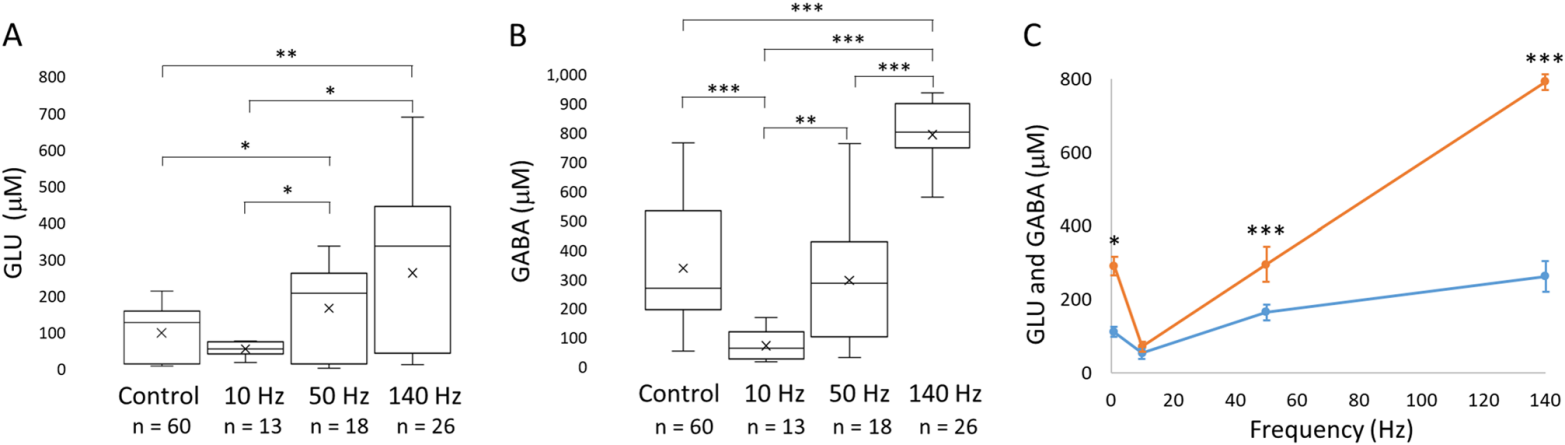
Peak GLU and GABA concentration rises with increasing stimulation frequency. The control stimulation was a single, 100-ms pulse, and the 10-, 50-, 140-Hz pulses were 5 s with 1-ms pulse widths. All stimulations were at 100-μA. The single, 100-ms control pulse resulted in higher mean peak levels of GLU and GABA than 10 Hz stimulation. (**A**) Mean peak GLU concentration increased as stimulation frequency increased from 10 to 140 Hz. (**B)** Mean peak GABA concentration also increased with stimulation frequency in a near-linear manner from 10 to 140 Hz. (For **A** and **B**, n denotes the total number of pulses.) (**C**) Lines on the scatter plot show that the rate of increase in extracellular GLU (blue) was lower from 50 to 140 Hz than from 10 to 50 Hz. The mean concentrations in response to the control pulse are displayed in the 0 Hz position. In contrast, extracellular GABA (orange) rose in a linear fashion with increasing stimulation frequency. Results are shown as mean ± SEM (error bars), n values shown on plots (**A**) and (**B**) are the same for (**C**). Statistically significant differences are denoted by asterisks over horizontal brackets in (**A**) and (**B**), and in (**C**), asterisks denote differences between GABA and GLU concentrations (independent Wilcoxon tests, *p ≤ 0.05; **p < 0.01; ***p < 0.001).

Peak concentration increased as stimulation frequency increased (Fig. 5). GABA concentration increased in a linear fashion, whereas GLU increased nonlinearly (Fig. 5C). GLU concentration increased to a lesser extent between 50 and 140 Hz than between 10 and 50 Hz, perhaps due to inhibition from increased GABA. Both GLU and GABA concentrations were higher after a single stimulation (100-ms control pulse) than after 10 Hz stimulation, although the difference for GLU was not significant (*p* = 0.08). The control pulse width was 100 ms for a single pulse, whereas the individual pulse width was 1 ms for the other stimulations over 5 s. As such, more current was delivered by the control pulse than the 10-Hz stimulation. GLU concentration increased significantly from 50 ± 5 µM to 166 ± 28 µM when the stimulation frequency was increased from 10 to 50 Hz. GLU concentration rose to 264 ± 43 µM (mean ± SEM) when the stimulation frequency was increased to 140 Hz, although the increase from 50 to 140 Hz pulses represented a nonsignificant trend (*p* = 0.063). However, the difference in concentration between 10 and 140 Hz stimulation was significant. Similarly, GABA concentration increased from 72 ± 14 µM to 296 ± 53 µM from 10 Hz to 50-Hz stimulation, and it rose further, to 793 ± 27 µM (mean ± SEM) in response to 140-Hz pulses. In addition, mean peak GLU and GABA concentrations were significantly different in response to the control pulse (Fig. 5C). Furthermore, the difference was highly significant for 50-Hz stimulation and for 140-Hz pulses.

**Figure 6:**
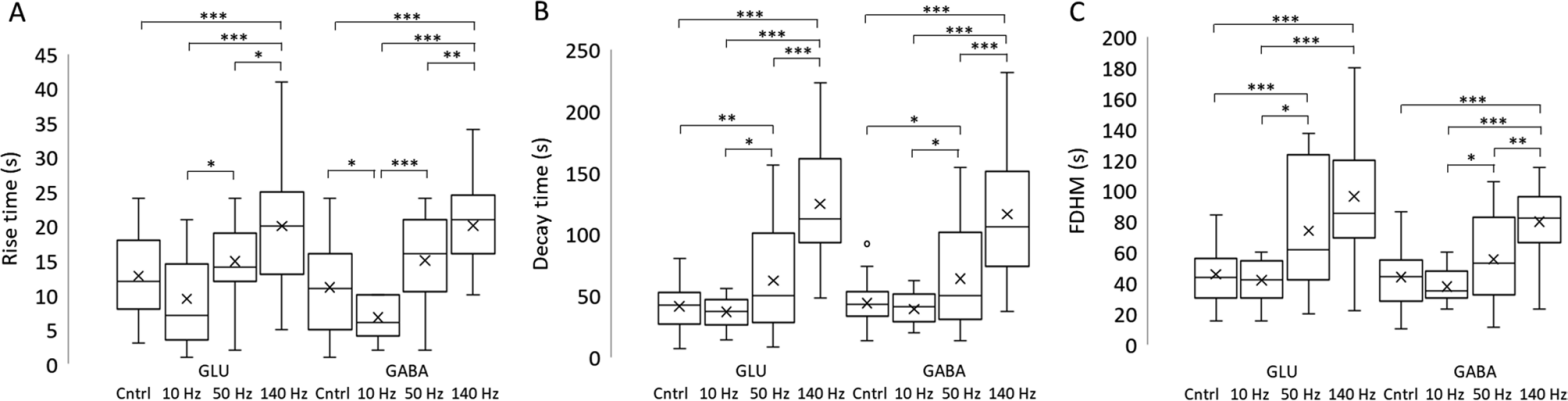
GLU and GABA peak characteristics. Rise time (**A**, T_R_), decay time (**B**, T_D_), and duration (**C**, FDHM) of extracellular GLU and GABA release increased as stimulation frequency increased. Although numerous significant differences were observed between 10, 50, and 140 Hz stimulations for release of each of the neurotransmitters, there were no significant differences between GLU and GABA for any one type of stimulation. (**A**) Differences in mean rise times between 10, 50, and 140-Hz for GLU and for GABA were significant. Control pulses had a mean rise time similar to 10 and 50 Hz pulses for GLU and 50 Hz pulses for GABA. (**B**) There were significant differences in decay time between all three stimulation frequencies. Control pulses had the same decay time as 10 Hz pulses. (**C**) Other than the similar FDHM for GLU between 50 and 140 Hz pulses, significant differences were observed between all other stimulation pulses for GLU and GABA. Control pulses had a mean FDHM similar to 10 Hz for GLU and close to 10 and 50 Hz pulses for GABA.*p < 0.05; **p < 0.01; ***p < 0.001 (The number of stimulations at each frequency is the same as shown in Fig. 5).

Dynamic features of extracellular GLU and GABA release, evident in the shape of the current peaks, scaled with stimulation frequency. For example, both the rise time (T_R_) and the decay time (T_D_) of GLU and GABA peaks increased as stimulation frequency increased (Fig. 6A, B). In general, the full duration at half maximum (FDHM) of the peaks (Fig. 6C) scaled with increasing stimulation frequency except for a non-significant difference between GLU peak duration after 50- and 140-Hz pulses. Interestingly, for the single control pulses, mean GLU and GABA T_R_ values were higher than the 10-Hz stimulation. Higher frequency stimulation may result in longer, repeated firing for some neurons and/or it may recruit neurons in a wider volume^54^. For the latter case, the neurotransmitters released by more distant neurons would take a longer time to diffuse toward the biosensors^55^ increasing the T_R_ and possibly FDHM. In the case of increased neurotransmitter release, it may require more time for full astrocytic uptake, which would increase the T_D_ and FDHM^38^. Alternatively, higher frequency stimulation may have resulted in greater release of GLU and GABA from astrocytes^56^ which could be relatively distant^57,58^. To demonstrate the effect of diffusion on rise time, we conducted an additional experiment in a beaker with three different stirring rates and an unstirred condition (Fig. S3). The rise time was seven to ten times longer for the unstirred versus stirred condition. No differences were observed between GLU and GABA for the same parameter and stimulation frequency.

### E/I ratio of excitatory glutamate and inhibitory GABA changes with stimulation frequency

**Figure 7:**
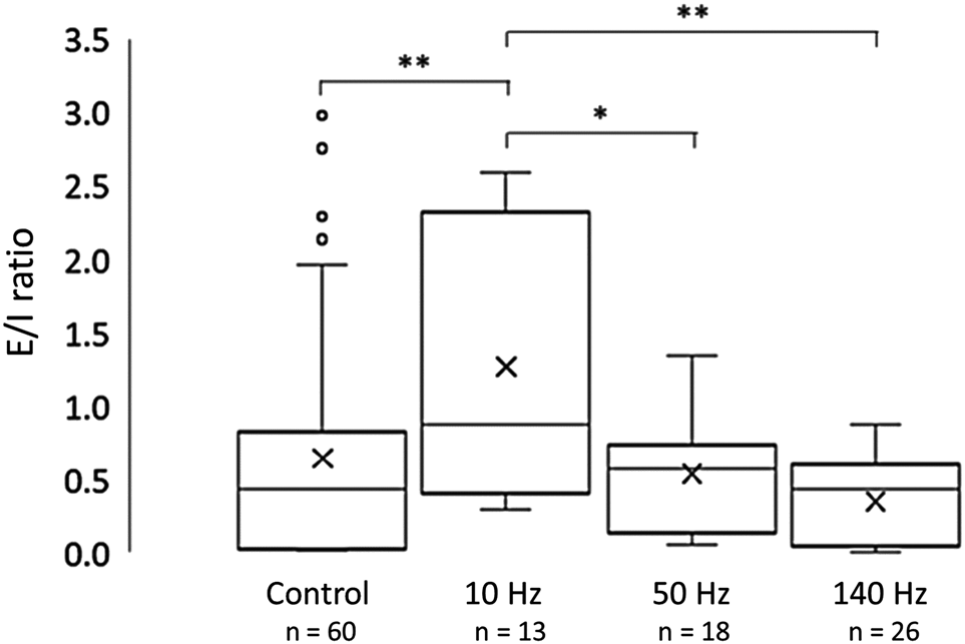
Excitatory vs inhibitory ratios. Ratio of peak concentrations of GLU (excitatory input, E) and GABA (inhibitory input, I). The E/I ratio decreased with increasing stimulation frequency. Significant decreases occurred between 10 and 50 Hz, and 10 and 140 Hz stimulation. A non-significant trend toward lower E/I values was observed for higher frequency stimulation between 50 and 140 Hz (*p* = 0.09). Wilcoxon rank sum test, *p < 0.05; **p < 0.01.

GLU is the major excitatory (E) neurotransmitter in the mammalian brain, and GABA is the major inhibitory (I) neurotransmitter. Both are plentiful in CA1. We calculated the ratio of GLU to GABA concentration (E/I ratio) for each stimulation condition (Fig. 7). Mean E/I ratios for a single control pulse and 10-, 50-, and 140-Hz pulses, were 0.64 ± 0.10, 1.27 ± 0.26, 0.54 ± 0.09, and 0.36 ± 0.06 (mean ± SEM), respectively. The reduction in E/I ratios as stimulation frequency increased was significant between 10- and 50-Hz, and 10- and 140-Hz pulses, and between control and 10-Hz stimulations. The reduction in the E/I ratio from 50 to 140 Hz stimulation was a non-significant trend (*p* = 0.09).

### In vivo recordings, 14 weeks apart, in the same rat shows distinct GLU and GABA signals related to behavior

**Figure 8:**
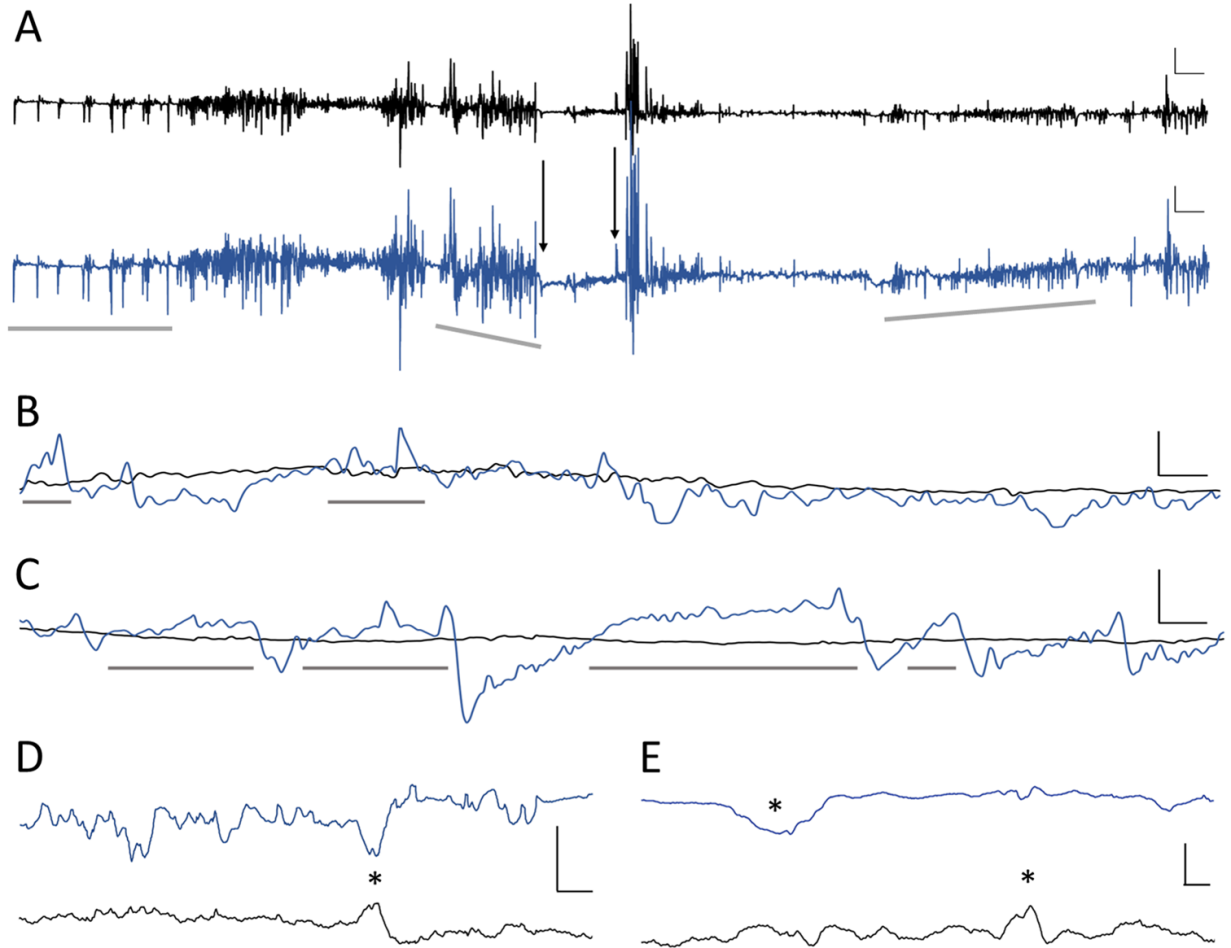
In vivo GLU (black) and GABA (blue) dynamics change with different types of behavior**.** Current shown in all panels is after subtraction of sentinel current for both channels and subtraction of GLU current from the GABA channel. (**A**) Several behaviors are captured in this 8-min 40-s recording. Interictal-like peaks appear in the first 70 s when the rat is not moving (horizontal gray bar). This is followed by walking and grooming (after the left gray bar and through the middle gray bar). Movement ceased between the two downward arrows as the rat abruptly stopped moving (frozen posture). A rapid decrease in GLU and GABA occurred at the onset of freezing (left arrow). GABA gradually increased until the point when the rat clearly exhibited seizure behavior with forelimb clonus, which is a Racine Scale 3 behavior during epileptic seizures in rats. Coinciding with clonus are brief sharp peaks in both neurotransmitters (right arrow). After this, GABA and GLU fluctuated rapidly, and after a few seconds the rat reared and fell, which are Racine Scale 4 and 5 behaviors, respectively. Maximum current fluctuations in both biosensor channels were observed just before and through rearing. After the rat ceased rearing, it continued to exhibit mouth and facial movements with some occasional head bobbing for the remainder of this recording segment. These are Racine Scale 1 and 2 behaviors, respectively^61^. During this period, GABA current dropped rapidly (just before inclined gray bar, **A**) and then steadily rose (slope denoted by inclined gray bar on right, **A**). Vertical scale bar denotes 1 nA and horizontal scale bar represents 20 s for both GLU and GABA traces. (**B**, **C**) Representative sleep–wake cycles at Wk 16 that illustrate the changes in GABA. GABA current steadily increased prior to each of six episodes of sleep (gray bars), during the rat’s light cycle, and remained elevated with some fluctuation during sleep. Immediately prior to waking, GABA levels rapidly fell and remained low during activity. Vertical scale bars denote 0.5 nA and 1.0 nA for top and bottom plots, respectively, and horizontal scale bars represent 2 min for both. (**D**) Representative recording two weeks after cannula implantation while the rat was walking in its home cage. Asterisk marks a concurrent decrease in GABA with an increase in GLU current. Vertical scale bar denotes 0.1 nA, and horizontal scale bar represents 125 ms. (**E**). Recording segment from the same rat, as shown in (**D**), eight weeks after the first recording. The rat was also walking in its home cage during this time period. Asterisks denote a decrease in GABA and an increase in GLU current; these events are separated by 600 ms. Vertical scale bar denotes 0.2 nA and horizontal scale bar represents 100 ms.

We recorded GABA and GLU in an awake, freely moving rat two weeks (Wk 2) after guide cannula implantation and again during Wk 10 and Wk 16, which were eight and fourteen weeks, respectively, following the first recording (Fig. 8). Freshly calibrated microwire biosensors were inserted into the permanently implanted cannula for each recording session. Between recordings, a filler wire was inserted into the cannula to maintain the patency of its lumen. After acquiring baseline recordings, we administered lithium and pilocarpine to initiate epileptogenesis. This is a model of temporal lobe epilepsy that is characterized by spontaneous seizures which begin a few weeks after drug administration. Current traces shown in Fig. 8 are after subtraction of sentinel current for both biosensor channels and also after subtraction of GLU current from the GABA channel. For better visualization, we did not include the sentinel channel; however, a plot with GLU, GABA and sentinel current is shown in Supplemental Data (Fig. S5). We also downsampled the recordings and used a moving average to smooth traces in Panels A–C to better visualize GLU and GABA baselines. GLU and GABA dynamics were markedly different between various behaviors, which included grooming, walking, sleeping, and epileptic seizures. We observed spontaneous seizures at Wk 10 and several sleep–wake cycles at Wk 16. We did not observe any movement artifacts when the rat walked, groomed, or slept. However, pronounced motion artifacts occurred whenever the rat bumped its head on the side of the cage. The magnitude of this artifact was greater than two times the highest current peaks seen during normal behaviors.

One example of how GLU and GABA vary with behavior can be seen in a segment of the Wk 10 recording (Fig. 8A). Activities during this recording include walking, resting, grooming, and several different types of seizure behaviors. At the start of this segment, the rat was awake but not moving (horizontal gray bar on the left). During this 70-s time period, several short and steep fluctuations occurred that resemble interictal spikes, commonly observed in electroencephalograms, that suggest a seizure has recently occurred or is about to occur^59,60^. The full duration of these spikes (time from departure to return to baseline) was 443.3 ± 136.6 ms (mean ± SD, n = 9) and the frequency of these spikes was 0.13 Hz. GABA and GLU concentrations were calculated using calibration curves for each biosensor that were constructed prior to recording. The concentration at these low points was 4 ± 1 μM and 2 ± 0.8 μM for GLU and GABA, respectively, and baseline levels were 18 ± 4 μM and 15 ± 5 μM, respectively (mean ± SD, n = 9).

The period of interictal-like spiking was followed by normal walking with larger and more frequent fluctuations in both neurotransmitters. After this behavior, we observed lower amplitude fluctuations on the same baseline as the rat rested. The fluctuation frequencies during these periods were similar (walking, 9.7 Hz; resting, 13.0 Hz). Following these time periods, rapid fluctuations in concentration (averaging 28 fluctuations/s, and varying by 6 ± 2 μM for GLU and 10 ± 3 μM for GABA) preceded and continued through a 21-s long period of grooming. A brief, 3-s rest period followed. During this time, the GABA baseline began to decrease while the GLU baseline remained unchanged. As the rat began grooming again, the same fluctuating behavior was observed. However, both GLU and GABA baseline concentrations steadily dropped from 18 μM to 8 μM for GLU and from 17 μM to 5 μM for GABA (Fig. 8A, middle gray bar shows GABA baseline slope). After a steep decrease in GABA (Fig. 8A, left downward arrow), baseline GABA slowly increased but remained below baseline by 50%, while baseline GLU remained steady.

Thirty-three seconds after the sudden drop in GABA, the rat had a seizure that began with forelimb clonus (Fig. 8A, right downward arrow) with brief simultaneous peaks in both neurotransmitters (GLU increased 50.0 ± 25.0% and GABA 50.0 ± 14.3% over their baseline concentrations, respectively). Forelimb clonus is a Racine Scale 3 behavior in rodent models of epilepsy^61^. Within two seconds, large amplitude and rapid changes in GLU and GABA began to occur. A few seconds later, the rat reared and fell over, which are Racine Scale 4 and 5 behaviors, respectively. The large peaks in both neurotransmitters ended as the seizure behavior transitioned to Racine Scale 1 facial movements. The baseline of both neurotransmitters remained below pre-seizure levels with progressively lower fluctuations in concentration of around 1 μM for each neurotransmitter. Baseline GLU concentration remained at 8 ± 4 μM while GABA slowly rose from 2 to 6 μM. Almost two min after the seizure began, the GABA signal suddenly decreased again to 2 μM and then began a steady increase over the next two minutes to 11 μM (Fig. 8A, inclined gray bar to right). Baseline levels of both neurotransmitters remained lower than pre-seizure baseline throughout the seizure.

We compared the mean baseline concentration for each behavior (Fig. 8A) and calculated an E/I ratio for each. As expected, seizure activity was dominated by excitation. The E/I for interictal activity was 1.20 and for Racine Scale 1–5 behaviors, it was 2.88. Conversely, awake behaviors of walking, resting and grooming were dominated by inhibition (E/I = 0.89). During the pre-seizure period (between arrows, Fig. 8A), and as the seizure wanned during the last two minutes of the recorded traces, the E/I ratio was 1.0. Notably, the mean E/I ratio for the entire period was dominated by excitation (E/I = 1.39).

A very different relationship of GLU and GABA signals was observed during sleep cycles. GABA signals rose before the onset of sleep. Prior to arousal, the GABA current rapidly declined. Six sleep–wake cycles from Wk 16 are shown in Fig. 8B, C, with sleep denoted by horizontal gray bars. Mean peak concentration of GABA during sleep increased 31.2 ± 8.6% (mean ± SD, n = 6) above the overall mean for the series of sleep–wake cycles and then decreased 31.9 ± 11.7% (mean ± SD, n = 6) during awake behaviors. Whereas relatively large changes were observed in GABA, the concentration of GLU remained stable throughout these cycles, although it did rise and fall during other types of behaviors (not shown) during the recording session. The maximum GABA concentration during periods of sleep and the minimum concentration for periods of wakefulness were used to calculate the E/I ratio for each episode. The mean E/I ratios for sleep and wakefulness were 0.67 ± 0.04 and 1.32 ± 0.24, respectively (mean ± SD, n = 6 each) indicating that inhibition dominated during sleep and excitation during wakefulness. Interestingly, the mean E/I for all sleep and wake periods was 0.995, which is close to complete balance.

Biosensor current was digitized at 1,000 Hz for all sessions. Recordings were generally down-sampled and smoothed to better observe shifts in neurotransmitter levels with behavior. When analyzing raw current data, subsecond fluctuations in GABA and GLU current were observed that were not related to electronic noise (i.e., not 60-Hz oscillation). Two examples are shown in Fig. 8 (Panels D & E); both recordings were acquired during calm walking. As glutamatergic and GABAergic neurons form feedback networks in the hippocampus, it was not surprising to observe some opposing transient changes in current. The first example illustrates this phenomenon with a decrease in GABA and corresponding increase in GLU (asterisk, Fig. 8D). This set of peaks had a FDHM of 70 ms. Peak current was determined using calibration curves for each biosensor channel. Mean GABA concentration before and after the peak was 3.8 μM and 5.1 μM, respectively. Concentration rapidly decreased by 0.6 μM then increased by 1.8 μM and remained slightly higher. GLU followed a similar time course, but the signal was inverted compared to GABA. Mean GLU concentrations before and after this peak were 15.3 μM and 12.1 μM, respectively. Peak concentration increased by 1 μM and then rapidly declined by 4 μM and remained low during this time segment. The second example of sub-second fluctuations in GABA and GLU current was recorded eight weeks later in Wk 10 (Fig. 8E). In contrast to the simultaneous, opposing peaks in Fig. 8D, the two peaks in Fig. 8E were separated by 600 ms. Mean GABA concentration before and after the drop (asterisk) was 19 μM and 22 μM, respectively. GABA rapidly dropped by 8 μM and then increased by 11 μM. Mean GLU concentration before and after the peak (asterisk) was 15 μM and 14 μM, respectively. GLU increased by 4 μM and decreased by 5 μM. The GABA and GLU peaks had a FDHM of 235 ms and 128 ms, respectively.

## Discussion

### Peak concentration varies with stimulation frequency

While extracellular GABA and GLU peak concentration in brain slices increased with increasing stimulation frequency, their responses were different. Mean peak GABA concentration in response to 140-Hz stimulation increased 26-fold over baseline compared to a 15-fold increase for GLU. In addition, we observed a linear increase in hippocampal GABA release with increasing stimulation frequency. This linear increase of GABA suggests the contribution of neurotransmitter from a non-neuronal source, such as astrocytes^56^. Up to one third of extracellular GABA in the hippocampus may be from non-vesicular neuronal and astrocytic release regulated by GABA transporters (GAT), especially GAT-1 and GAT-3^25^. In contrast, GLU increased at a lower rate from 50- to 140-Hz stimulation versus from 10- to 50-Hz. Our results are in agreement with Mantovani et al., who reported a marked increase in GABA release and little effect on GLU release in human neocortical brain slices after 130-Hz stimulation^41^. Using optogenetics, Chiang et al. also found that high-frequency stimulation of the hippocampus predominantly drove GABA release^42^. In addition, increasingly higher levels of GABA could have exerted an inhibitory effect, reducing activation of glutamatergic neurons^62^. Synaptically released GABA provides much of the inhibition in the hippocampus. However, Boddum et al. reported on another mechanism of GLU inhibition in the hippocampus that relies not on synaptically-released GABA, but on GAT-3 transporters on astrocytes^63^. GAT-3 activity releases ATP/adenosine which inhibits release of GLU via presynaptic adenosine receptors. Thus, these three mechanisms would likely limit the increases in GLU release.

Peak GABA concentrations in our study were much higher than commonly reported concentrations in the hippocampus using microdialysis. Those studies reported basal levels up to 0.5 μM for GABA^37,64^ and, after KCl stimulation, levels increased up to 2.5 μM for GABA^64^. In contrast, at least one microdialysis study reported a nearly 1,100% higher concentration after stimulation with KCl^25^. In addition, another research group demonstrated that synaptically released GABA in cultured rat primary hippocampal cells reach transiently high concentrations (~ 1.5 to 3 mM) using electrophysiology and computer modeling^65^. Similar to GABA, the baseline and peak GLU concentration that we observed was higher than microdialysis studies^37,64^. However, our range of concentrations were within the range of concentrations found in other studies^64,66^. In the present study, we stimulated the Schaffer collaterals, which is likely to elicit synchronous firing in CA1 that recruits a relatively large number of glutamatergic pyramidal cells and GABAergic interneurons^53^. Given the placement of our microwires, a large proportion of our biosensor surfaces were probably in close contact with active synapses of these cells^66,^ not only during the response to electrical stimulation, but also between stimulation as these neurons are known to fire tonically^67^. Thus, we would expect to detect higher baseline and peak concentrations of GABA and GLU after electrical stimulation.

### E/I ratio varies nonlinearly with stimulation frequency ex vivo

We observed a marked decrease in the E/I ratio in CA1 from 10- to 50-Hz stimulation and from 10- to 140-Hz pulses. This decrease was nonlinear. There was also a nonsignificant trend suggesting a possible decrease from 50- to 140-Hz. For the low frequency, 10-Hz stimulation, the E/I ratio was greater than one, suggesting that 10-Hz pulses elicit an overall excitatory response in CA1. In contrast, the 50- and 140-Hz stimulations resulted in E/I ratios of less than one. Even though both GLU and GABA concentrations increased with 50- and 140-Hz pulses, the balance shifted to an inhibitory response with markedly higher GABA concentrations, especially after 140-Hz stimulation. Increasing GABA concentration likely limited the increase in GLU, which switched the balance from excitatory to inhibitory dominance^15^. The lower sensitivity of glutamatergic neurons in the hippocampus to high-frequency stimulation^41,42^ along with the robust response of GABAergic neurons to high-frequency stimulation^68^ also likely contributed to the shift in the E/I ratio. While the ratio decreased with increasing stimulation frequency, the concentration of both neurotransmitters increased with increasing frequency. To avoid GLU excitotoxicity, the absolute concentration of GLU and GABA may not be as important as the ratio of excitatory to inhibitory signaling.

### Uses of stimulation to treat neurological disorders

The motivation for selecting our three stimulation frequencies was driven by clinical experience where 140 Hz stimulation is often used in deep brain stimulation (DBS) therapy against a seizure or tremor, 50 Hz stimulation is used in direct cortical stimulation (DCS) to induce seizure, while 10 Hz stimulation is used in transcranial alternating current stimulation (tACS) therapy. Durand’s group showed seizure suppression by high-frequency optogenetic stimulation in hippocampus of animal models of epilepsy, where the suppression was attributed to the activation of GABA receptors in interneurons^42^. This work and others have demonstrated that high-frequency electrical stimulation suppresses seizures by generating a depolarization block related to the inactivation of sodium channels or driving GABA release during stimulation. In addition, it has been estimated that a single GABAergic cell may affect more than a thousand pyramidal cells^69^. Therefore, activation of GABAergic neurons becomes dominant at high-frequency stimulation, which has led to seizure suppression. In our work, we observed a huge activation of GABAergic neurons at 140 Hz. This excess GABA could have reduced excitatory synaptic transmission by decreasing excitatory postsynaptic potentials and GLU release and even block action potential propagation as suggested by Ruiz et al.^70^. We observed progressively larger peak concentrations of GABA and GLU as stimulation frequency increased. However, the E/I ratio was not linear with frequency, suggesting that GABA and GLU release in response to different frequencies of stimulation is complex. Our probe system should be useful in studying responses to a wide range of stimulation parameters.

### In vivo GLU and GABA dynamics change during different types of behavior

During in vivo recordings across fourteen weeks, we observed markedly different baseline levels and frequency of fluctuations in GABA and GLU concentrations. We also identified changes in the E/I ratio for different types of behaviors, including walking, resting, grooming, seizing, and sleeping. Furthermore, these dynamic features often occurred on a subsecond time scale, as seen in seizure activity, while others occurred over several minutes, such as in sleep–wake cycles. The recordings also revealed changes in E/I ratio throughout the course of a long seizure and in sleep–wake cycles. In addition, we demonstrated that a freshly calibrated microwire array inserted into an implanted cannula could be used to acquire signals through a wide range of behaviors, suggesting that this system is suitable for longitudinal studies.

In epilepsy, brief paroxysmal electrical discharges, called interictal spikes are frequently observed in electroencephalograms in between seizures. Spike duration is under 250-ms, and they occur at low frequency, with several seconds between spikes^59,60^. Interestingly, we observed interictal-like patterns in GLU and GABA levels a few minutes prior to an epileptic seizure. These had the same low-frequency pattern with a slightly longer mean peak duration of 433 ms. The longer duration of the neurotransmitter spikes compared to electrographic spikes could derive from slower kinetics of neurotransmitter release from astrocytes and/or uptake^25^ and diffusion from more distant neurons^54^. The concurrent and sudden decrease in GABA and GLU with a rapid rebound to baseline of the interictal-like spikes warrants further study^59,60^. It is commonly reported that neurons fire in abnormal levels of synchrony during a seizure^71^. We also noticed that large fluctuations in concentration occurred at the same time, starting with interictal spiking and continued through the seizure even though the baseline levels of the two neurotransmitters changed independently from each other. All five Racine Scale behaviors were evident during the recording, and for each one, the amplitude and frequency of the variation were distinct. In addition, baseline GABA decreased almost two minutes prior to the seizure, which was expected^46,72^. Furthermore, GABA and GLU increased just before the seizure, as others have shown^71^. GABA remained lower than baseline, as expected^47,^ which likely contributed to the high E/I ratio. Used in longitudinal studies, these microwire biosensors could help reveal the role of timing and levels of GLU and GABA in epileptogenesis, in seizure propagation, and the effects of therapeutic brain stimulation and drugs.

In contrast to the high-frequency changes in GLU and GABA levels that occurred during seizure and grooming, a 2-h period of recording that included several sleep episodes exhibited long, slow increases and decreases in GABA. Even though our biosensor detected higher concentrations of the two neurotransmitters than microdialysis, the changes in GABA concentration and relatively stable baseline GLU concentration during sleep and arousal are consistent with microdialysis studies in small mammals. These studies have shown that GABA concentration is significantly higher during sleep^43–45,73^. Additionally, microdialysis studies have discovered that GLU concentration does not change significantly during this cycle^45,73–75^. Interestingly, the mean E/I ratio was 0.995 through all recorded sleep–wake cycles, which was well-balanced. Our observations demonstrate what others have shown, which is that excitatory and inhibitory signaling are normally balanced at multiple time scales^7^. Seizures and interictal spiking are associated with a temporary disruption in this balance^7,72^. This temporary condition was reported in a human and nonhuman primate study, which showed that the E/I balance was restored, even after seizure activities^7^.

We observed rapid increases and decreases in both neurotransmitters during our in vivo recordings. One example of this was the large, subsecond, synchronous changes in GLU and GABA during putative interictal spiking prior to a seizure. However, all of our recordings also exhibited subsecond changes in neurotransmitter levels that were asynchronous or that were closely timed. This is consistent with an electrophysiology study showing the close relationship of excitatory and inhibitory signaling that maintained their balance over multiple time scales^7^. Brief as our peaks were, their time scale was about an order of magnitude longer than electrographic spikes. Thus, our recordings are not directly comparable to electrophysiology studies. However, it would be useful to simultaneously record electrical and neurochemical signals to determine if the electrical activity is closely correlated with these rapid fluctuations in neurotransmitter levels. Whether and when they differ could provide greater insights into the effects of neuronal and non-neuronal changes in glutamatergic and GABAergic tone and excitotoxicity.

### Probe geometry is suitable for applications from in vitro to in vivo studies

Our microwire biosensor design is not only advantageous for future, longitudinal in vivo recording, its geometry has distinct advantages over shank-style neural recording probes for brain slice and cell culture models. In our previous studies, we experienced difficulties in using shank-style microbiosensor arrays when recording from brain slices^34^ and cultured cells^38^. Shank-style probes are designed to record electrical signals from different depths (on the order of mm) of the brain for in vivo studies. In contrast, brain slices are cut thin (on the order of a few hundred μM) and placed horizontally in a recording chamber to extend tissue viability and function. Only some of the sensor sites on most shank style probes fit into the tissue. The others are unusable. Thus, a geometric profile in which all of the microwire biosensor sites contact the slice in the same horizontal plane provides a significant advantage. Similarly, cultured cells for in vitro models grow in a thin layer (~ 10 μM) on a flat cell culture dish. Again, a microwire biosensor in which all probe sites are in a horizontal plane equidistant from the cells is a desirable arrangement. Thus, our microwire biosensor offers advantages for in vitro, ex vivo, and in vivo recording.

Fabrication of microwire biosensors with 127-μm diameter wire is performed in-house without the need for micromachining. Producing these biosensors in-house offers several advantages. (1) The mPD layer can be coated on the outside for longer sensor life for in vivo applications, or on the inside for higher sensitivity, short-term use^76^. (2) Additional wires can be added and placed at different depths for recording in more than one brain area. (3) Pt microwires are relatively inexpensive and easy to coat with a micropipettor compared to high-density shank arrays. (4) It is relatively easy to change the geometrical area of the biosensors by simply changing the Pt microwire diameter. (5) A high-density probe (up to 8 biosensors) can be assembled with thinner wires to create a probe diameter of less than 300 microns. This should result in minimal inflammation, which is a desirable biosensor characteristic for in vivo recordings. The ease of handling the microwire biosensor with the microwires banded together provided the strength and stability to insert it into a cannula for real-time in vivo recording. The ability to remove the biosensor and replace it with a freshly calibrated device facilitated a long-term pilot study of GLU and GABA dynamics with high temporal resolution in the hundreds of ms. This improvement in temporal resolution, combined with the ability to record from multiple regions in the rodent brain, will enable researchers to study dynamics of GLU and GABA signaling on a subsecond time scale for the first time.

### Conclusion

Our microwire biosensor recorded GABA, GLU, and interferent signals in real time in a brain slice model and in the hippocampus of a live rat in three recording sessions over fourteen weeks. Changes in E/I and the pattern of fluctuations in neurotransmitter levels during sleep, awake behavior, and epileptic signaling were consistent with reports in the literature^7,15,43,45,47,59^. Furthermore, we detected significant differences in GABA and GLU concentration between different stimulation parameters in brain slices. These concentrations were consistent with observations reported in a range of other studies^41,42^. Moreover, we observed a significant shift in the E/I ratio from excitatory to inhibitory dominance as stimulation frequency increased^41,42,77^. This shift was nonlinear, and it suggests that GABA release is a component of a homeostatic mechanism that exists in the hippocampus to prevent excitotoxic damage. The ability to monitor these dynamic changes in vivo, with subsecond time resolution periodically over several months, will make this GLU and GABA microwire biosensor an important tool for exploring the effects of stimulation and drugs on neuronal networks and for expanding our understanding of physiological processes in health, disease, injury, and aging.

## Methods

We have developed a platinum (Pt) microwire biosensor probe that is easily fabricated in a lab that has electrochemistry expertise. Our biosensor probe is composed of three Pt microwires to detect GABA, GLU, and interferents, respectively. The GABA biosensor is based on a novel process recently developed in our lab that does not require the addition of substrates, which makes it easier to use for in vitro^38^ and ex vivo applications^34,^ and which enables in vivo recordings^38,62^. Additionally, when the ends of the novel microwire biosensors are in the same plane, as we report here, the geometry is suitable for placement into cell culture dishes and brain slices. It is also optimal for in vivo recording in brain regions with laminar structure, such as the cerebral cortex and hippocampus in animals.

### Microwire biosensor fabrication

In the first step, Teflon coated, 127-µm diameter Pt wire (#77300, AM-systems, Sequim, WA, USA) was cut into 4-cm lengths with a PXC058 tube cutter (Coilhouse Pneumatics, East Brunswick, NJ, USA). One cut end served as the recording side. On the other, non-recording side, a 1-cm length of Teflon coating was gently stripped away using a steel scalpel blade. This end was wrapped around a miniature electrical connector pin (#3128-4-00-15-00-00-08-0, Mill-max connector, Oyster Bay, NY, USA) at a later step. The exposed Pt microwires were electrochemically cleaned using the cyclic voltammetry (CV) method. The CV was run in a 2-electrode setup using an Ag/AgCl reference/counter electrode in a 0.05 M H_2_SO_4_ electrolyte solution, and the potential was cycled between − 0.3 V to + 1.0 V with a 20 mV/s scan rate for 15 cycles^76,78^. The rationale for cleaning the microwire is to increase hydrogen peroxide sensitivity, which is due to significant reductions in the charge transfer resistances of the Pt microelectrode grains and grain boundaries (details not shown). After the microwire cleaning, they were heated in an oven at 65° C for 20 min. The Pt microwires were then calibrated to measure their sensitivity towards H_2_O_2_, the electroactive by-product of the microbiosensor (Supplemental Fig. S1). This initial calibration helped to ensure the three different channels (GABA, GLU, and sentinel) have the same sensitivity towards H_2_O_2_. The microwires were stored dry at room temperature in a clean plastic container until ready for the enzyme coating.

After the completion of the H_2_O_2_ calibration, enzyme solutions were drop-cast using a micropipettor onto the recording end of the Pt microwires (details of drop casing procedure are in Hossain et al., 2018)^34^. Briefly, a fresh enzyme matrix solution was prepared that contains bovine serum albumin (BSA, 1%) and the crosslinker, glutaraldehyde (0.125%), in DI water. We employed the crosslinking method to achieve good stability by retaining the enzyme structure and functionality. This solution was mixed with 0.1 U/mL GOx for the GLU microbiosensor and a mixture of 0.1 U/mL GOx and 0.1 U/mL GABASE for the GABA microbiosensor. The third microwire, called the sentinel channel, was constructed by applying only the enzyme matrix solution with BSA and glutaraldehyde (i.e., the matrix does not contain any enzymes). The sentinel microsensor plays an important role in measuring the presence of small electroactive molecules (interferents) present in the vicinity of the biosensor probe that can be easily oxidized at the + 0.7 V applied to all three Pt microwires^76^. Examples of common interferents are nitric oxide, dopamine and AA. In our previous study, we concluded that our externally applied mPD layer prevents most of the AA from reaching the Pt microelectrode and, thus, contributed a small current^34^. The sentinel channel was instrumental in subtracting the current from these interferents. The enzyme-coated microwires were stored in the dark at room temperature for 48 h. Once the enzyme was cross-linked with the enzyme matrix, a size-exclusion layer of mPD was electrochemically coated onto all three coated microwires. This provides selectivity or specificity to the microbiosensor by preventing the diffusion of the interferents, primarily AA, that are present in high concentrations (up to millimolar range)^79^. A 10 mM mPD solution was prepared in 1 M NaCl and then purged with nitrogen for 30 min before use. The mPD was coated by cycling between + 0.2 V and + 0.8 V at a scan rate of 50 mV/s for 100 cycles, using a saturated calomel electrode as a reference and counter electrode. The details of this coating procedure are in Hossain et al., 2018^34^. After coating, the freshly prepared microbiosensors were stored in a dry, dark cool place until they were assembled into a probe. We originally stored our biosensors at room temperature, however, we found that storing them in 4 °C gave greater sensitivity towards GABA^62^.

To form the microwire biosensor, we tightly wrapped the uncoated, exposed “other” end of the Pt wire of each biosensor around a 500-μm diameter, gold-coated Mill-Max connection pin to form a secure physical and electrical contact. Care was taken not to wrap the Pt wire too tightly around the pin to avoid breaking the wire. The contact region was then covered with heat shrink wrap (HS-TBG 3/64" 2:1 CL, TE connectivity, Schaffhausen, Switzerland) to provide seamless electrical contact between the biosensor and the pins on the Mill-Max connector (Fig. 1, dagger). The Mill-Max connector connects to the potentiostat cable for recording. Next, the coated ends of the three microwires were bound together with smaller diameter heat shrink wrap (asterisk in Fig. 1 and inset, #103-0246, Nordson Medical, Salem, NH, USA). The shrink wrap provided protection of the thin Teflon wire coating against abrasion, and it placed the wires in close proximity to each other, holding the ends of the wires in the same recording plane. It also added rigidity to the microwire probe for improved handling during experiments. A barbed Leur lock cap (51525K125 nylon quick-turn barbed plug 3/16″ inside diameter, McMaster-Carr, USA) was used to secure the biosensor to a permanently implanted guide cannula with a male Luer lock fitting. The microwires were inserted through the opening in the cap so that the end of the microwire bundle would protrude 100-μm beyond the end of a permanently implanted guide cannula for in vivo recording. For our system, this length was 4.0 cm. A Teflon-insulated Ag/AgCl wire was also inserted through the cap to connect to the reference electrode integrated into the cannula. Six-cm of insulation was stripped from the end of the wire, and then the wire was twisted into a loop around the Luer lock cap so that the stripped portion will make a secure electrical contact with a similar loop of Ag/AgCl wire integrated into the connector of the cannula. The remainder of the Ag/AgCl wire was threaded through the cap and cut 0.3 cm above the top end of the cap. This end of the wire was stripped and soldered to a male pin connector (520,200, A-M Systems, Sequim, WA, USA). We secured the wires into the cap using dental acrylic (Ortho-Jet BCA, Lang Dental Manufacturing Company, Inc., Wheeling, IL, USA). Electrical continuity for each biosensor channel was confirmed using a FAST-16MkIII potentiostat (Quanteon, LLC, Nicholasville, Kentucky, USA).

### Animal care and brain slice preparation

Male Sprague–Dawley rats were housed on a 12 h light/dark cycle. Food and water were provided ad libitum. All procedures were approved by the Louisiana Tech University Institutional Care and Use Committee and were in accordance with the Guide for the Care and Use of Laboratory Animals. Euthanasia was performed in accordance with the AVMA Guidelines on Euthanasia.

Male Sprague–Dawley rats were briefly anesthetized with 5% isoflurane gas and euthanized by guillotine. Brains were quickly extracted, placed in cold artificial cerebrospinal fluid (aCSF), and cut into 500-μm thick sections. Hippocampal slices were then placed in an incubation chamber filled with room-temperature aCSF bubbled with carbogen (5% CO_2_, 95% O_2_). Individual slices selected for recording were placed in a perfusion chamber (Scientific Systems Design, Inc.) continuously supplied with aCSF at 37 °C and bubbled with carbogen. Waste products were continuously suctioned away using the facility’s vacuum system and a 500-ml flask^38^.

### Microwire guide cannula fabrication

Each guide cannula was constructed from a 20-gauge stainless steel hypodermic needle with a plastic Luer lock fitting (BD305125, Becton, Dickson and Company, Franklin Lakes, NJ, USA). The hypodermic needle was blunt cut and sanded to a length of 2.8 mm, which allowed for its insertion into the hippocampus at the desired depth. A 25-gauge hypodermic needle (BD305175, Becton, Dickson and Company) was fashioned into a filler wire for the lumen of the guide cannula. The filler wire keeps blood and tissue from infiltrating into the guide cannula. To facilitate the insertion of the filler wire into the cannula, most of the plastic Luer lock fitting was removed using a Dremel Multipro with a circular 3.4-cm diameter cutting bit, leaving approximately a 1–2-mm section of the plastic fitting that surrounds the top of the needle. A 1-mm hole was drilled into the center of a Luer lock screw cap (51525K311 nylon quick-turn cap, McMaster-Carr). The needle was passed through the hole in the cap until it reached the remaining portion of the plastic fitting. Cyanoacrylate adhesive was used to secure the needle to the cap. The needle was cut to a length that filled the guide cannula but did not protrude through it. For our guide cannula, the filler wire was cut to 2.8 mm in length. Needles were cut with a Dremel 3.4-cm diameter cutting bit and the ends were smoothed with a flat mill file of fine coarseness. An Ag/AgCl reference electrode was attached to the outside and parallel to the metal portion of the guide cannula using cyanoacrylate adhesive. The implanted end of the reference electrode was positioned at the end of the cannula. The connector end of this electrode was fashioned into a loop and pressed flat against the top of the Luer lock fitting. The insulation on the loop portion was removed to facilitate contact with the microwire biosensor for recording. When not recording, the filler wire cap covers the loop to prevent damage to the wire.

### Cannula implantation

A cannula, with the filler wire inserted, was implanted into the CA1 region of the hippocampus, following a protocol approved by the Louisiana Tech University IACUC. To reduce stress, Male Sprague–Dawley rats were lightly anesthetized using isoflurane before an intramuscular injection of 75 mg ketamine HCl and 0.25 mg dexmedetomidine HCl per 1 kg of animal weight. Using aseptic techniques, a 3.5-mm diameter craniotomy was made 5.0 mm posterior from Bregma, 2.3 mm right lateral to the midline, and 2.8 mm deep measured from the top of the skull. Four 1.50-mm stainless-steel anchor screws (Stoelting Co.) were implanted bilaterally along the outer edge of the scalp incision and a fifth screw located just anterior to lambda and 2.0 mm laterally to the right of the sagittal suture as anchors for the implant. The cannula, with the filler wire inserted, was attached to a custom, 3D printed holder on a stereotaxic frame and lowered at 100-μm per min to the desired depth. The cannula and screws were secured using dental acrylic (Ortho-Jet BCA) which was applied to completely cover the bone screws, encapsulate the lower part the guide cannula fitting, and seal the exposed skull. Skin incisions were closed with interrupted sutures, and topical powder (Neo-Predef, Zoetis) was applied for antibiotic, anti-inflammatory, and analgesic effects. After this, the rat was given an intramuscular injection of 1 mg/kg Atipamezole hydrochloride to bring the animal out of anesthesia and then monitored until it regained sternal recumbency. Rats were housed individually after surgery. Animals were given at least two weeks to heal before recording.

### Amperometric calibration and recording

We used an eight-channel FAST-16mkIII potentiostat with a 2-electrode configuration, including an Ag/AgCl reference electrode. We applied + 0.7 V across the biosensor electrodes, which is optimal for H_2_O_2_ detection. Calibrations were carried out in a 40 mL phosphate buffered saline solution (PBS) in a 50-ml glass beaker. Analytes, such as GABA, GLU and AA, were introduced into the solution with a syringe pump (Legato 100 syringe pump, KD Scientific, USA) to obtain desired concentrations (μM). Solutions were maintained at 37° C and continuously stirred at 200 rpm. All current measurements were repeated 6 times (n = 6). FAST analysis software (Quanteon, LLC) was used to analyze some data. Sensitivity toward the analyte (GABA or GLU) was defined as the change in current per unit of analyte addition, which was slope (pA/μM) of the calibration curves. To calculate the current density (nA μM^–1^ cm^–2^), the slope was divided by the Pt microwire area (1.27 × 10^–4^ cm^2^). The limit of detection (LOD) was derived by dividing the standard deviation of the baseline (3 times the standard deviation of 10 points from the baseline when no analyte was present) by the least squares slope. These were calculated using the method described by the FAST system manufacturer (2014 software manual, Quanteon, LLC). One-way ANOVA was performed to compare differences between conditions. Significance was defined as *p* < 0.05. Error values on plots are shown as mean ± SEM. When justified by ANOVA, 2-tailed Students t-test were performed for pairwise comparisons.

A calibration was performed for each biosensor 20–30 min prior to its use for slice recording experiments. The two main purposes for this additional calibration are to ensure adequate functionality of the probe after storage and to have the most recent sensitivity values for GLU and GABA.

### Stimulation system and brain slice recording

A home-built, Arduino-based signal generator (Supplemental Fig. S4) and a stimulus isolator (A365, World Precision Instruments, USA) were used to deliver monophasic, 100-μA current pulses of varying frequencies to a paired tungsten stimulation electrode. Four types of pulses were programmed and verified with an oscilloscope. Three types of 5-s pulse trains with 1-ms pulse width were programmed to deliver 10 Hz, 50 Hz, and 140 Hz stimulation, and a single, 100-ms control pulse was also programmed^18,23,49–51^.

Two micromanipulators (M3301, World Precision Instrument, Hessen, Germany), an upright brightfield microscope (Eclipse E600FN, Nikon, Japan), and a custom, acrylic stage facilitated placement of the microwire biosensor in the pyramidal layer of hippocampal region CA1. Paired tungsten wire stimulation electrodes, 65-μm radius, were placed within the Schaffer collaterals, ~ 150 μm from the microwire biosensor, as previously reported^34,53^. A fresh biosensor was used each day. After the response current returned to baseline from 10-, 50- or 140-Hz stimulation, we delivered a single 100-ms control pulse to assess the health of the slice and to verify that stimulations were not inducing potentiation^53^. When the response current to a single pulse reached 80% or less than the mean response of the first three control pulses, the slice was discarded. Current was continuously recorded at a sampling rate of 1,000 Hz using the FAST system.

### In vivo recording of GLU and GABA in awake, freely moving rat

Prior to recording, a rat was anesthetized in a chamber using 5% isoflurane for 5 min. Immediately after removing the rat from the chamber, the filler wire was removed from the guide cannula, and a sterile microwire biosensor array was inserted into the guide cannula. It was secured by twisting the Luer lock cap on the microwire array (Fig. 1B); this also ensured contact with the reference electrode. (The filler wire was stored in 91% isopropyl alcohol during surgery. After recording, it was dried with a sterile tissue and reinserted.) The pin connector on the microwire biosensor array was inserted into the FAST potentiostat system, and the reference wire connector pin was inserted into a separate channel on the potentiostat fitting. The rat was placed in a standard, clear plastic housing cage without a cover for recording. Corn cob bedding (Envigo, Madison, WI, USA) was used to line to bottom of the cage. Two to three pieces of dry chow and a water bottle were placed in the cage. A + 0.7 V bias voltage was applied to the microwire biosensors to facilitate oxidation of H_2_O_2_. The FAST system was set to acquire current data at 1,000 Hz. We used the Fast Analysis software, which is integrated into the FAST recording system to store time and current data in 1,000-s segments as csv files for offline analysis. After recording, the microwire biosensor was removed, and a clean filler wire was inserted into the cannula. For subsequent recordings, we used freshly calibrated biosensors.

### Model of temporal lobe epilepsy

Two weeks or longer after implantation of the cannula for housing the biosensor, rats were given 3 mmol/kg lithium chloride (Li-Cl-, Sigma-Aldrich, USA) in 0.9% sterile saline (Teknova, USA) intraperitoneally (ip). Twenty-two hours later, 30 mg/kg pilocarpine (Sigma-Aldrich, USA) in 0.9% sterile saline (Teknova) was administered subcutaneously to induce status epilepticus. Status epilepticus initiates epileptogenesis, resulting in spontaneous recurrent seizures a few weeks afterward^80^. Status epilepticus began approximately twenty minutes after pilocarpine injection. Seizure behaviors, including Racine scale 1–5, were documented^61^. After 3 h, an antiepileptic cocktail of phenobarbital and diazepam (25 mg/kg and 10 mg/kg, respectively) were administered ip to improve survival^81^. Spontaneous recurrent seizures developed within a few weeks^80^.

### Identification of responses to stimulation and definitions of peak parameters

**Figure 9:**
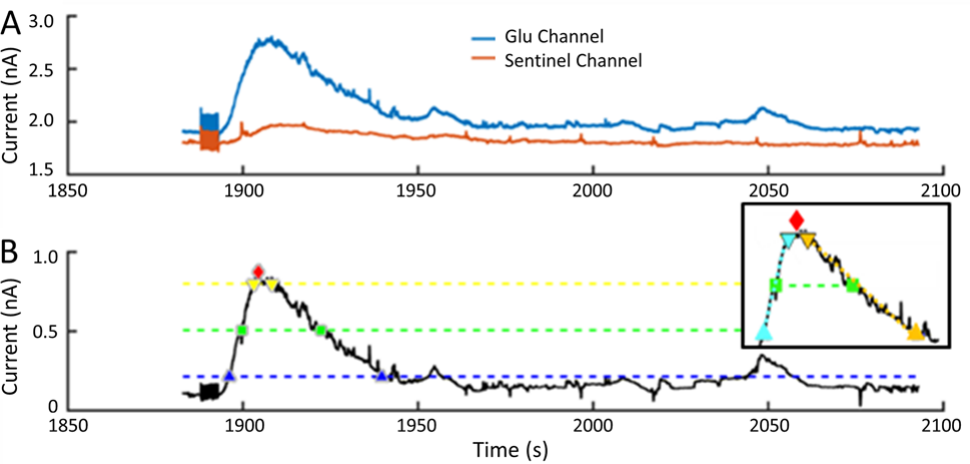
Example plot of current response to electrical stimulation (only GLU and sentinel channels are shown for better visualization of details). (**A**) Raw current recorded from the GLU and sentinel channels. (Stimulation artifact begins at 1890 s). (**B**) Resulting GLU current after the sentinel channel is subtracted from the raw current of the GLU channel. The red diamond indicates the maximum value of the response to stimulation. The blue, green, and yellow dashed lines indicate the thresholds for 10%, 50%, and 90% of the maximum current, respectively. The blue, green, and yellow markers indicate where the glutamate signal crossed the dashed thresholds; these were the time points used to calculate rise and decay times and pulse width. **Inset (B)** Response parameters: The responses to stimulation were compared using four parameters of the peaks (all in seconds), including maximum current (peak, red diamond), rise time (T_R10-90_, time between blue triangles), decay time (T_D90-10_, time between orange triangles), and full duration at half maximum (FDHM, time between green squares). The dotted lines connecting symbols denote the time segments of the respective parameters^38^.

Current data were imported into Matlab for analysis. Individual responses were isolated based on noted stimulation times. The current from interferents on the sentinel channel was subtracted from the GLU channel to derive the current due to GLU. An example plot is shown in Fig. 9 with the raw signals for the GLU and sentinel channels in Panel A and the subtracted GLU signal (I_GLU_ − I_Sentinel_) in Panel B. Response current was allowed to return to baseline before each subsequent stimulation; the mean of the last 10 s of each response was used to define that response’s baseline. Maximum, 10%, 50%, and 90% values of the peak response were then determined (illustrated in Fig. 9B, adapted from Scoggin et al.^38^. The time between 10 and 90% of the rising peak were used to determine the rise time (T_R_), and the time between 90 and 10% of the falling peak were used to calculate the decay time (T_D_). The time between the rise and fall of the peak at 50% of the maximum current was used to measure the FDHM. Responses were discarded if features were indistinguishable from noise (< 2X noise) or if the sentinel signal increased above the GLU channel. GABA current was obtained by subtraction of both the sentinel and GLU channel current. Peak values were converted into μM concentration based on calibration curves acquired 30 min prior to recording.

### Statistical analysis

Results for each treatment condition were expressed as the mean and standard error of the mean (SEM). Error bars in figures represent the SEM. Initial evaluation of peak concentration values indicated that they were not normally distributed. The independent Wilcoxon test was used to compare means with significance at α = 0.05.

## Supplementary information

Supplementary Information.

## Data Availability

Data are available at https://drive.google.com/drive/folders/13wGHc1Ky0LZKNe8aI8QQfSzqBq5VoZut?usp=sharing.
