# Supplementary material for: Novel microwire-based biosensor probe for simultaneous real-time measurement of glutamate and GABA dynamics in vitro and in vivo
Source: Sci Rep. 2020 Jul 29;10:12777. doi: 10.1038/s41598-020-69636-1 (PMC7392771; doi:10.1038/s41598-020-69636-1)
Supplement: Supplementary file 1 — Supplementary Information. [file 41598_2020_69636_MOESM1_ESM.pdf]

Supplemental Data

Novel microwire-based biosensor probe for simultaneous real-time measurement of glutamate and GABA dynamics in vitro and in vivo

P. Timothy Doughty[1,†], Imran Hossain[2,†], Chenggong Gong[2], Kayla A. Ponder[1], Sandipan Pati[3], Prabhu U. Arumugam[1,2,*], Teresa A. Murray[1,*]

[1] Center for Biomedical Engineering and Rehabilitation Sciences, Louisiana Tech University, Ruston, LA USA; [2] Institute for Micromanufacturing, Louisiana Tech University, Ruston, LA USA; [3] UAB Epilepsy Center/Department of Neurology, The University of Alabama at Birmingham, Birmingham, Alabama, USA)

[†] Authors contributed equally to this work. * Corresponding authors contributed equally to the work: Dr. Arumugam, parumug@latech.edu; Dr. Murray, tmurray@latech.edu

Sensitivity of microwire biosensor channels to H_2O_2

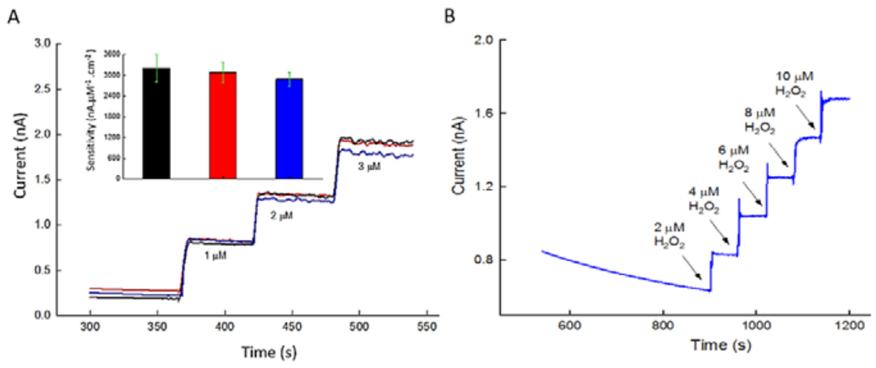

Figure S1. (A) The response of the three microwire biosensor channels, GLU (red), GABA (black), and sentinel (blue), to H_2O_2, prior coating with mPD, rises linearly as H_2O_2 is added. **Inset.** All three channels had similar sensitivities towards H_2O_2, as expected. Histogram bars are the mean sensitivity and error bars denote ± SEM, bar colors correspond to sites shown in plot (ANOVA p = 0.39). (B) Sentinel channel response to H_2O_2 after mPD coating (arrows show the injection of H_2O_2). Sensitivity is 748±89 nA/µM.cm2, which is almost 70% less than our CV cleaned wire.

Selectivity of biosensor to GABA and GLU, effective block of ascorbic acid

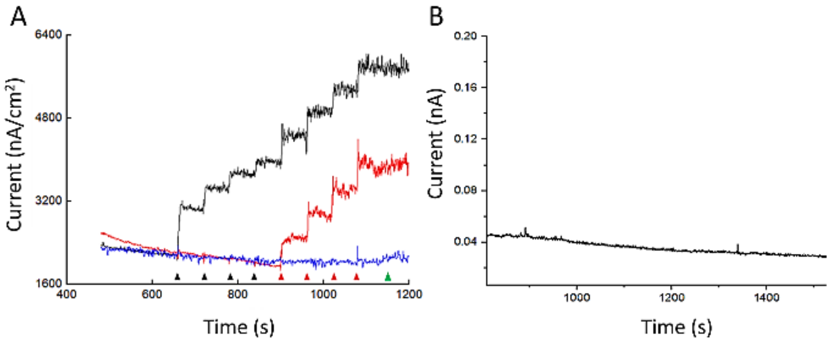

Figure S2. Selectivity of biosensor channels. (A) A representative recording on GABA (black curve), glutamate (GLU, red curve) and sentinel (blue curve) channels in terms of current density. GLU and sentinel channel currents were unaffected by the addition of GABA (20 µM steps, black arrowheads). GLU channel current increased with each addition of GLU (10 µM steps, red arrowheads). GABA channel current increased with each addition of GABA and of GLU, as expected because this biosensor has both GABASE and glutamate oxidase. Addition of 100-µM ascorbic acid (green arrowhead) did not evoke a current above noise, which is expected due to its mPD coating. Current was measured (+0.7 V vs Ag/AgCl) in 1 mM α-ketoglutarate, stirred at 200 rpm, at 37° C. (B) GLU channel in presence of 1mM α-ketoglutarate shows minimal change in current with time of 0.1 pA per minute in this channel compared to a 1 pA per minute increase in the GABA channel.

Effect of stirring rate on rise time

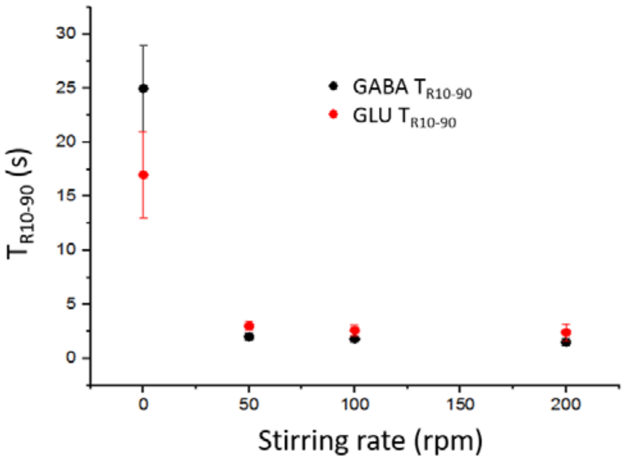

Figure S3. Effect of stirring rate on GABA and GLU rise time ($T_{R10\text{-}90}$). As convection is forced with higher stirring rate, the $T_{R10\text{-}90}$ decreases (n = 3). Notably, $T_{R10\text{-}90}$ is markedly higher when the solution is not stirred, showing the effect of diffusion on $T_{R10\text{-}90}$. Diffusion can be even slower in brain slices given the tortuosity of brain tissue.

Arduino controller

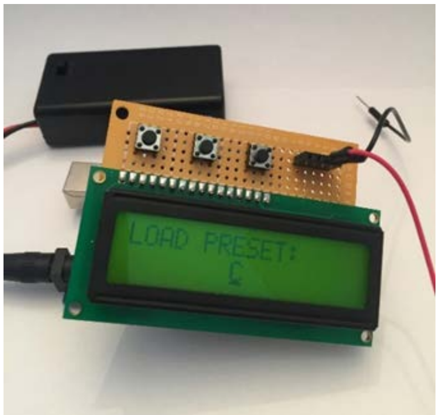

Figure S4. Arduino controller for delivery of electrical stimulation. Four different pulse parameters were programmed (control, single 100-ms pulse; and 5-second pulse trains at A, 10 Hz; B, 50 Hz; and C, 140 Hz). Pulses A, B, and C were randomly selected for experiments. The control pulse was delivered between each frequency stimulation. Photo shows the controller set for stimulation C (140 Hz).

Our MATLAB m files are available on the MATLAB File Exchange at https://www.mathworks.com/matlabcentral/fileexchange/.

Recording showing sentinel channel

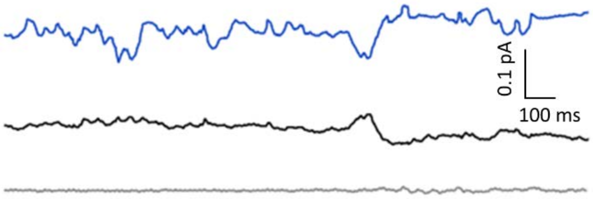

Figure S5. Segment of in vivo recording from Fig. 8, main text, showing all three channels (raw 1000-Hz recording). The sentinel channel is unprocessed data (raw signal, gray). GLU (blue) and GABA (black) traces are net current.
